# Soothing Ointments and the Indications for Their Use

**Published:** 1882-02

**Authors:** 


### Soothing Ointments and the Indications for their Use.

The oleates, comprising Crocker's zinc oleate, Martindale's lime oleate, and Sawyer's lead oleate, constitute undoubtedly a very valuable addition to our means of healing inflamed surfaces and soothing irritable skins, and they are very justly coming into extensive use. Dr. McCall Anderson has lately proposed a bismuth oleate of which the following is the formula, and to be prepared in a similar manner to the well-known zinc oleate. ℞ Bismuthi oxidi, ℥i; acidi oleici, ℥viij; ceræ albæ, ℥iij; vaseline, ℥ix; olei rosæ, ♏v; M. et. ft. ung.—*The Specialist.*

